# The Etiology of Alcoholic Degeneration

**Published:** 1877-08

**Authors:** 


### ARTICLE V.

### *The Etiology of Alcoholic Degeneration.*

Almost weekly, the exchanges we receive contain reports of cases where the complications brought about by alcoholism add largely to the danger of the patient. The inven tion of processes of distillation has led to a widespread introduction of distilled liquors where formerly fermented beverages were almost solely consumed. Thus statistics from the cider and wine districts of France and Germany show a growing use of beetroot brandy and similar form of spirits.

Some have maintained that the injurious effects of alcoholism arise more from fraudulent mixtures and the addition of poisonous ingredients, such as strychnia, cocculus indicus, aloes, fuchsine, etc., than from alcohol itself. Unwise and not over-scrupulous advocates of the temperance cause have frequently seized upon these alleged adulterations as arguments for their orations; and the tables have been turned upon them by dealers who guarantee "pure and unadulterated liquors only," and by non-temperance writers, who have maintained that such genuine alcoholic beverages are harmless even in considerable quantities. Thus, as is always the case, the men who advocate what is not true in order to do good have hurt their position and the reputation of their side.

In fact, wines and liquors in this country very rarely contain any injurious adulteration. The "manufactured" champagnes, brandies and liquors, so common at the bars of saloons, are made from "high wines" diluted, colored with burned sugar, or other innocuous substance, sweetened and flavored with very small quantities of essential oils. It is safe to say that no strychnine, cocculus indicus, alum, copperas, picric acid, or other deleterious substance, is used at all. All such statements emanate from the inventive brains of unscrupulous temperance orators.

The evidence for the truth of what we have just said is very ample given in the second Annual Report of the Inspector and Assayer of Liquors to the Commonwealth of Massachusetts, Professor James F. Babcock. He presents, in this Report, about four hundred analyses of all varieties of ordinary bar drinks, and did not find among them all any poisonous adulteration whatever. Of course the new whiskies had more or less fusel oil, but this, though deleterious, is no added product. Of this standard American drink he says:

"Many vile epithets, as 'benzine' and 'Jersey lightning' have been bestowed upon whisky, and perhaps deservedly, for new whisky is very different in its qualities from old; and it is to new or raw whisky that these opprobrious titles are applied. Whisky is rarely, if ever, adulterated with any injurious substances. Raw or new whisky owes its harsh flavor and burning taste not to substances intentionally added as adulterants, but to the presence of small portions of fusel oil and other bodies naturally produced by the fermentation, and which, by age or exposure to the oxidizing influence of the air, are either removed or changed into more agreeable products. Whisky, such as is used for the manufacture of alcohol, is almost strictly pure, as there is no incentive whatever to its adulteration.

"Whisky filtered several times through charcoal, which removes all coloring matter and other impurities, is undoubtedly the very purest form of liquor to be obtained, and though less agreeable, perhaps, to the palate, it is believed by many to be fully equal, for all medicinal purposes to the very best brands of imported spirits."

Professor Babcock also examined, with care, nine samples of lager beer and ale sold at retail stores in Boston. They came from different breweries in and near Boston and New York. In their analysis, the processes Dragendorff and Otto-Stas were employed for the examination of alkaloids and fraudulent or injurious bitters. The results showed that all of the samples were absolutely free from *coculus indicus*, *picric acid*, *strychnia*, or any other bitter, except that of hops and malt. All the samples were found to be pure, and free from injurious substances as alcohol in any form can ever be.

This conclusion is the same that has been reached elsewhere. The results of very numerous analyses of various kinds of beer, by chemists in this country and in Europe, have failed to show the presence of strychnia or other injurious adulteration in any of the samples examined.

Prof. C. F. Chandler, of the New York Board of Health, examined five different varieties of beer, upon which he reported—

"A most thorough examination failed to reveal any indication of the presence of picric acid, picrotoxine (the peculiar principle of *cocculus indicus*,) of alum, copperas, or any adulteration whatever."

The inference is fair, therefore, that the symptoms of alcoholism are owing to alcohol, and not to other poisonous ingredients contained in alcoholic beverages.—*Med. and Surg. Reporter.*

